# Inspiratory Lung Expansion in Patients with Interstitial Lung Disease: CT Histogram Analyses

**DOI:** 10.1038/s41598-018-33638-x

**Published:** 2018-10-15

**Authors:** Junghoan Park, Julip Jung, Soon Ho Yoon, Jin Mo Goo, Helen Hong, Jeong-Hwa Yoon

**Affiliations:** 1 Department of Radiology, Seoul National University Hospital, Seoul, Korea; 2 Department of Radiology, Seoul National University College of Medicine, Seoul, Korea; 3 Department of Software Convergence, Seoul Women’s University, Seoul, Korea; 4 Institute of Radiation Medicine, Seoul National University Medical Research Center, Seoul, Korea; 5 Interdisciplinary Program in Medical Informatics, Seoul National University College of Medicine, Seoul, Korea

**Keywords:** 3-D reconstruction, Image processing, Respiratory tract diseases, Biomarkers

## Abstract

This study aimed to evaluate inspiratory lung expansion in patients with interstitial lung disease (ILD) using histogram analyses based on advanced image registration between inspiratory and expiratory CT scans. We included 16 female ILD patients and eight age- and sex-matched normal controls who underwent full-inspiratory and expiratory CT scans. The CT scans were sequentially aligned based on the surface, landmarks, and attenuation of the lung parenchyma. Histogram analyses were performed on the degree of lung expansion (DLE) of each pixel between the aligned images in x-, y-, z-axes, and 3-dimensionally (3D). The overall mean registration error was 1.9 mm between the CT scans. The DLE_3D_ in ILD patients was smaller than in the controls (mean, 17.6 mm vs. 26.9 mm; p = 0.023), and less heterogeneous in terms of standard deviation, entropy, and uniformity (p < 0.05). These results were mainly due to similar results in the DLE_Z_ of the lower lungs. A forced vital capacity tended to be weakly correlated with mean (r^2^ = 0.210; p = 0.074), and histogram parameters (r^2^ = 0.194–0.251; p = 0.048–0.100) of the DLE_3D_ in the lower lung in ILD patients. Our findings indicate that reduced and less heterogeneous inspiratory lung expansion in ILD patients can be identified by using advanced accurate image registration.

## Introduction

Idiopathic pulmonary fibrosis (IPF), the most common form of interstitial lung disease (ILD), is a distinct type of chronic, progressive, fibrosing interstitial pneumonia of unknown cause, predominantly occurring in older adults^1–3^. The overall prognosis of IPF remained poor until the last few years, with a median survival ranging from 3 to 5 years and a 5-year survival rate ranging from 30% to 50%^4,5^. There had been no proved treatment other than lung transplantation for IPF. However, recent studies proved that nintedanib and pirfenidone could delay the progression of the fibrotic process in IPF, subsequently improving the overall prognosis. The use of these anti-fibrotic drugs is currently recommended for the treatment of IPF^2,6^.

Chest computed tomography (CT) is a key component of the management of ILD, as it is useful for the diagnosis, stratification of severity, assessment of treatment response, and prognosis prediction. Indeed, subjective evaluations of the degree of fibrosis on chest CT scans have been found to be useful for assessing severity and disease progression in IPF patients^7,8^. However, substantial variability existed in the subjective assessment across readers^9^. The objective quantification of severity and disease progression in ILD patients is becoming increasingly important.

Three-dimensional respiratory lung motion — in other words, the degree of respiratory lung inflation — is quantifiable by image registration between inspiratory and expiratory CT scans^10^. It was reported to be a useful option for assessing pulmonary functional loss in cases of chronic obstructive lung disease^11^. However, it has not yet been reported in patients with ILD, presumably due to the difficulty of accurate image registration of fibrotic lungs between inspiratory and expiratory CT scans. Recently introduced advanced image registration algorithms using attenuation- and landmark-based registration have shown to be allow more accurate registration in both animal and human study^10^. This algorithm may give a chance to overcome the difficulty of accurate image registration of fibrotic lungs during the respiration in patients with ILD. Thus, the purpose of our study was to evaluate inspiratory lung expansion in patients with ILD using histogram analyses based on advanced image registration of inspiratory and expiratory CT scans.

## Results

### Patient population

A total of 24 patients (mean age, 59.6 ± 10.2 years; age range, 41–76 years), encompassing 8 normal female controls (mean age, 58.8 ± 11.1 years; age range, 42–73 years) with normal CT scans, and 16 age- and sex-matched patients with ILD (mean age, 60.0 ± 10.1 years; age range, 41–76 years) who underwent paired full inspiratory-end expiratory thin-section chest CT scans were included. Male patients were excluded since there were no normal male subjects. Among the 8 controls, 5 had connective tissue disease and 3 were healthy subjects. Among the 16 patients, 10 were diagnosed as having connective tissue disease-related ILD (4 cases of systemic sclerosis, 3 cases of dermatomyositis, 2 cases of mixed connective tissue disease, and 1 case of rheumatoid arthritis), 4 as having IPF, and 2 as having nonspecific interstitial pneumonia (NSIP). Three patients had a pathologically proven diagnosis by means of surgical lung biopsy, and other diagnoses were made through a multi-disciplinary discussion between an experienced pulmonologist and radiologist.

### Accuracy of image segmentation and registration

The Dice similarity coefficient (DSC) showed a good intra-observer agreement between the repeated lung segmentation (mean DSC, 0.972; range, 0.814–1.000). The detailed data of intra-observer agreement was shown in Supplementary Table 1.

The mean registration errors without image registration and with surface-based affine registration were 13.1 ± 6.7 mm and 15.6 ± 8.7 mm, and 8.2 ± 6.0 mm and 7.5 ± 6.5 mm in the right and left lung, respectively. When attenuation-based deformation was additionally applied without landmark-based registration, the error was 5.7 ± 4.7 mm and 7.7 ± 7.4 mm, respectively. The errors further decreased to 4.1 ± 2.9 mm and 4.8 ± 2.9 mm, respectively, by applying a bronchial landmark, and the application of both bronchial and peripheral vascular landmarks finally resulted in errors of 1.9 ± 1.2 mm and 1.9 ± 0.9 mm in the right and left lung, respectively (Supplementary Table 2).

In terms of lung volume, the mean volume difference between inspiratory and expiratory lung volume was 484.2 ± 206.9 cm^3^ and 421.4 ± 229.5 cm^3^ in the right and left lung before registration, respectively. The volume difference was sequentially decreased with applying affine, landmark-based, and deformable registration, and the volume difference between inspiratory and registered expiratory lung volume became 91.6 ± 81.3 cm^3^ and 99.9 ± 93.6 cm^3^ in the right and left lung after all registrations were applied, respectively (Supplementary Table 3).

### Inspiratory lung expansion analysis

The degree of fibrosis of the whole lung on visual analysis was 17.2% ± 14.8% in ILD patients. The degrees of fibrosis in the upper, middle, and lower lungs were 8.1% ± 13.9%, 10.3% ± 13.1%, and 33.1% ± 21.6%, respectively. The degree of fibrosis in the lower lung was significantly higher than in the upper and middle lungs in ILD patients (p = 0.001).

**Figure 1:**
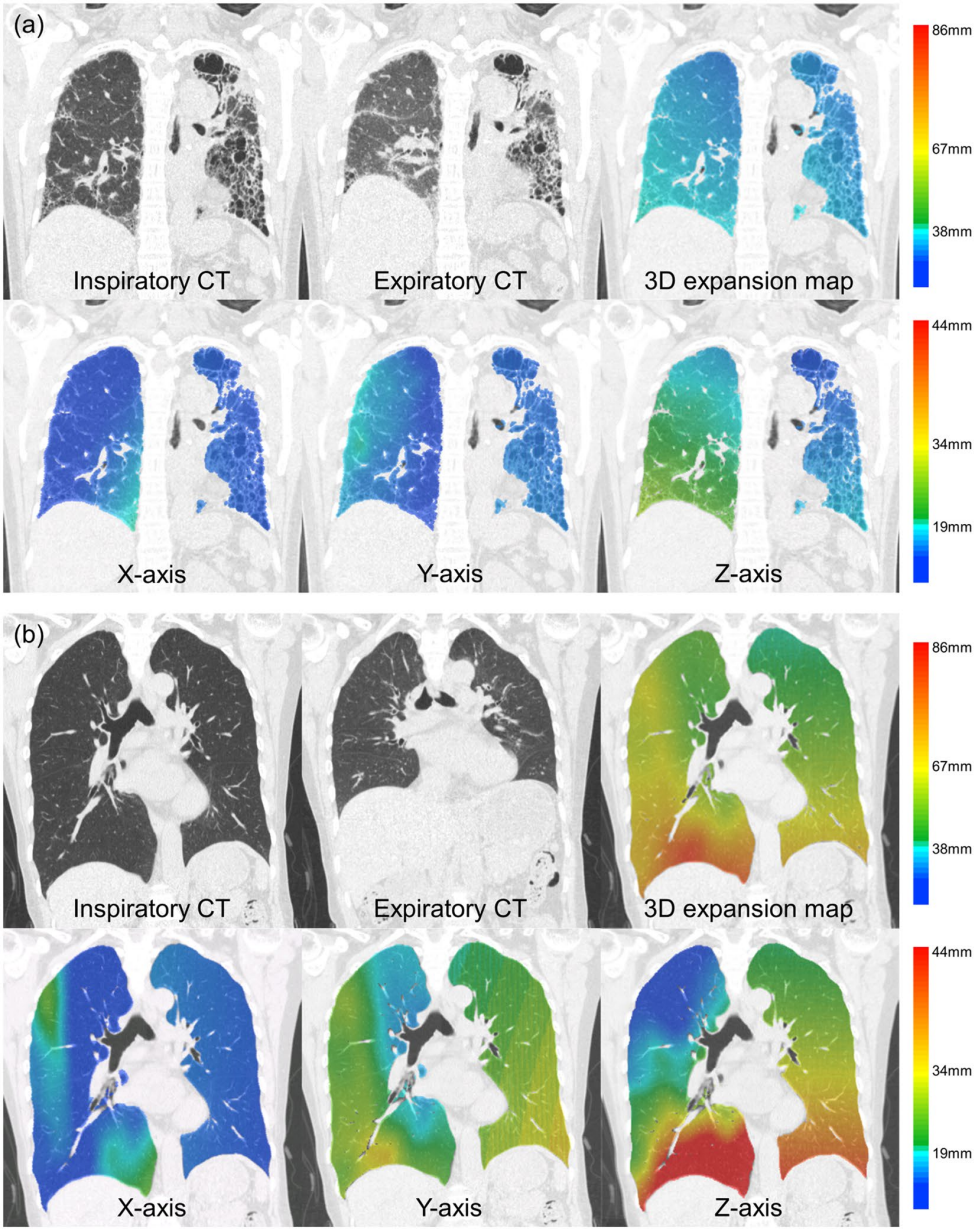
Representative images of paired full-inspiratory, full-expiratory CT images and lung expansion map in 3D and x-, y-, z-axis in (**a**) interstitial lung disease patient and (**b**) normal control.

**Figure 2:**
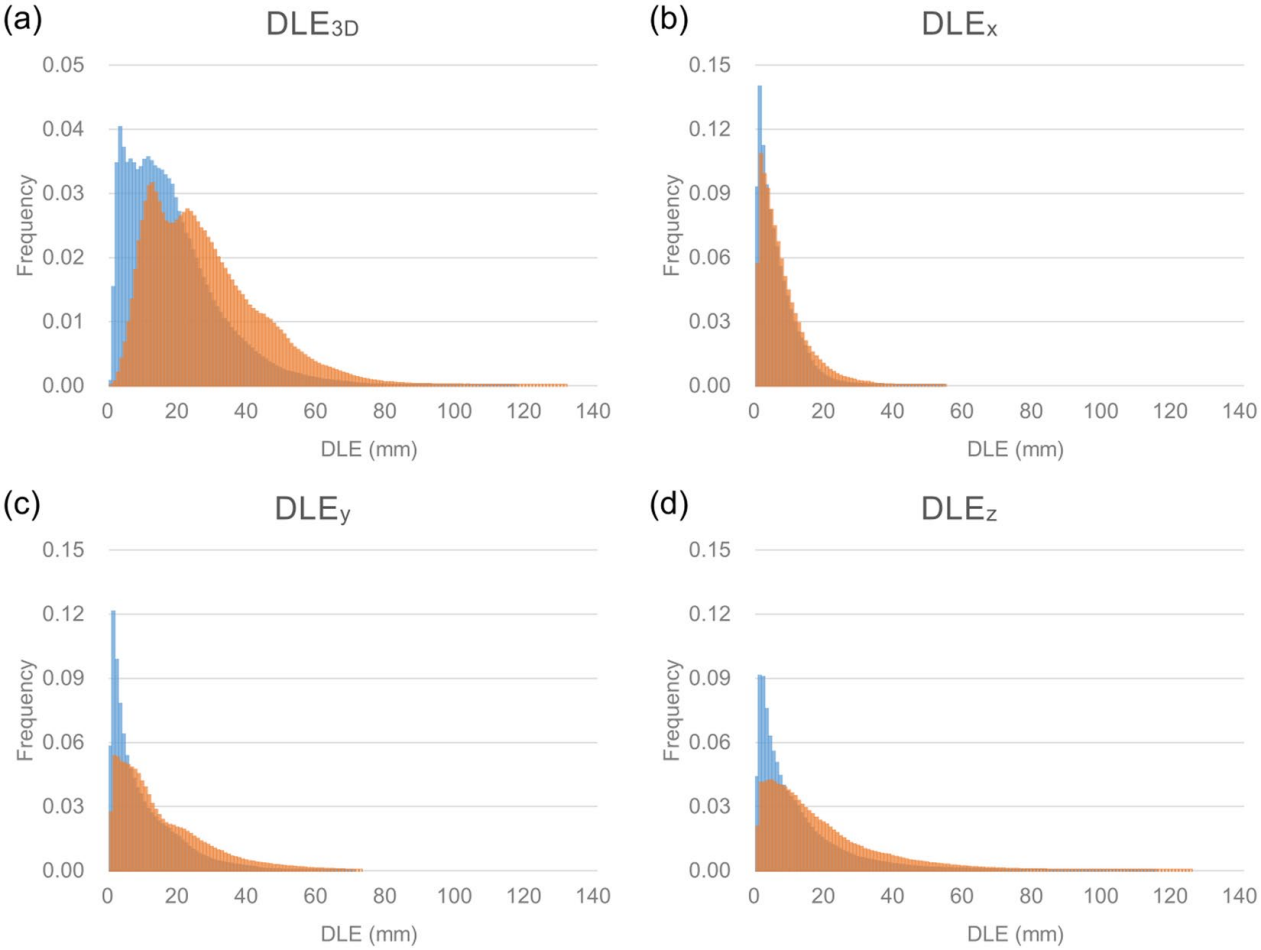
Histogram of degree of whole lung expansion in patients with interstitial lung disease patients (blue) and normal control (orange) in (**a**) 3-dimensional, and (**b**) x-, (**c**) y-, (**d**) z-axes.

**Figure 3:**
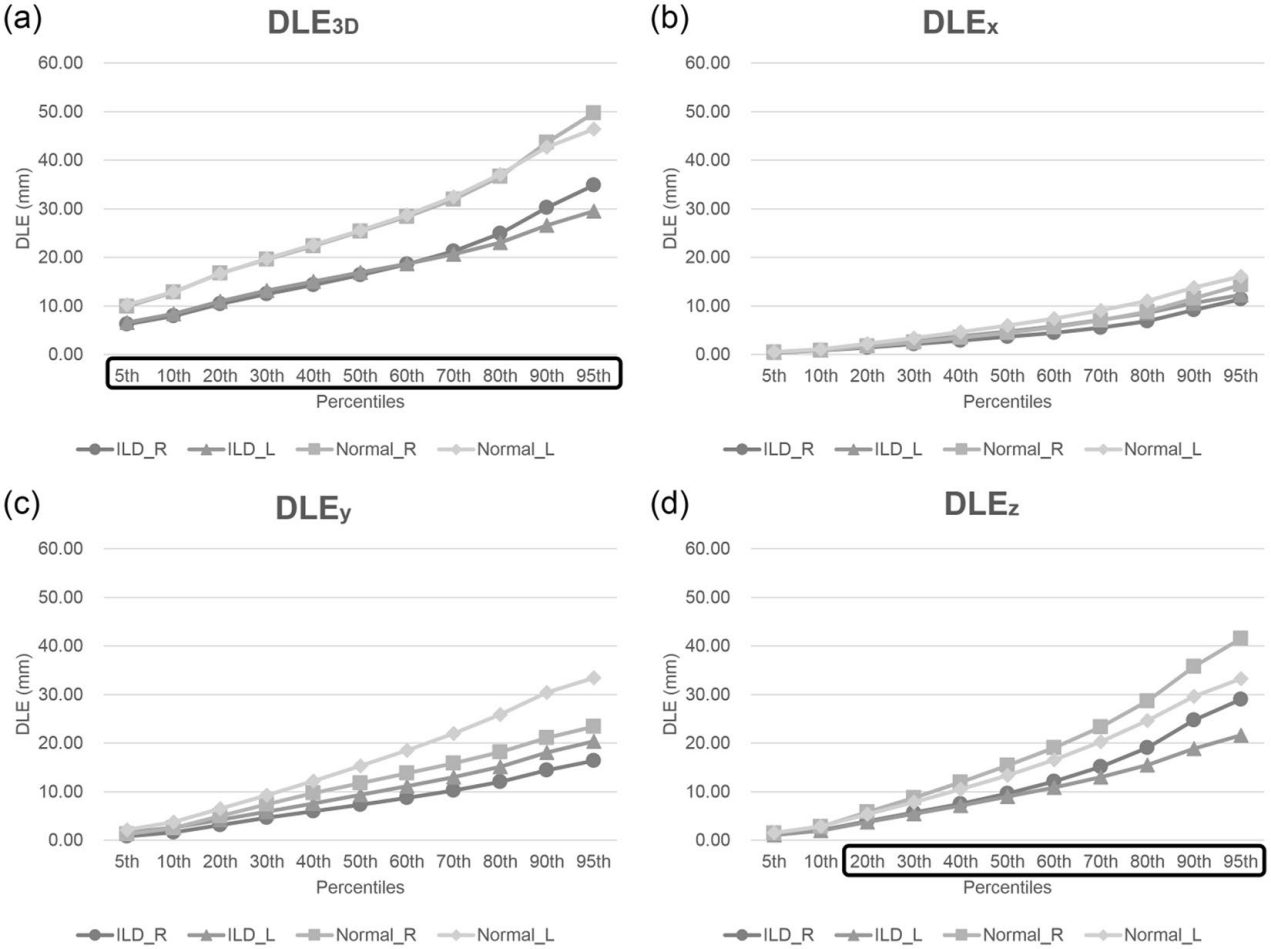
Graphs representing 5^th^ to 95^th^ percentile values of degree of whole lung expansion (**a**) 3-dimensionally, and in the (**b**) x-axis, (**c**) y-axis, (**d**) z-axis. A box with a black line indicates a statistically significant difference between two groups (p < 0.05).

Degree of lung expansion (DLE) of each pixel between the full-inspiratory and expiratory CT scans was measured in horizontal axis (x-axis; DLE_x_), ventrodorsal axis (y-axis; DLE_y_), craniocaudal axis (z-axis; DLE_z_), and 3-dimensionally (3D; DLE_3D_) (Reply to the grammar issue 4). Regarding whole lung expansion, the mean DLE_3D_ of ILD patients was significantly smaller than those of normal controls (17.6 mm vs. 26.9 mm, p = 0.023) (Table 1 and Fig. 1). The histogram of ILD patients had significantly higher kurtosis (p = 0.016) than that of normal controls and tended to be more positively skewed (p = 0.235) (Fig. 2). Statistical significance was found in the 5th to 95th percentiles of DLE_3D_ (all p < 0.05) (Fig. 3 and Supplementary Table 4). The DLE_3D_ values were more homogeneous in ILD patients than in normal controls, as shown by a smaller standard deviation (p = 0.015), less entropy (p = 0.018), and greater uniformity (p = 0.015) (Table 1). Among the three axes, similar results were found in the histogram parameters of DLE_z_, with statistically significant differences between the two groups for mean (p = 0.011), standard deviation (p = 0.021), skewness (p = 0.012), kurtosis (p = 0.003), entropy (p = 0.013), uniformity (p = 0.005), and 20th to 95th percentiles. A similar trend was also observed in the y-axis, but none of the histogram parameters showed statistically significant differences (Table 1).

**Table 1:** Baseline characteristics, histogram parameters of DLE in ILD patients and normal controls.

		ILD (n = 16)	Normal control (n = 8)	P-value
Age (years)		60.0 ± 10.1	58.8 ± 11.1	0.881
Height (m)		1.55 ± 0.06	1.56 ± 0.06	0.700
Degree of fibrosis (%)		16.3 ± 14.4	—	
PFT	FVC (L)	2.01 ± 0.50	2.95 ± 0.51	0.013
	%FVC (%)	76.1 ± 23.8	103.2 ± 8.8	0.005
	DLCO (mL/mmHg/min)	9.52 ± 1.56	15.32 ± 2.43	0.005
	%DLCO (%)	56.9 ± 13.3	85.6 ± 17.5	<0.001
	VA (L)	2.90 ± 0.44	3.94 ± 0.30	<0.001
Histogram parameters				
DLE_3D_	Mean (mm)	17.58 ± 9.34	26.87 ± 9.90	0.023
	Standard deviation (mm)	7.95 ± 3.91	11.73 ± 4.24	0.015
	Skewness	0.74 ± 0.48	0.57 ± 0.35	0.235
	Kurtosis	3.71 ± 1.19	3.17 ± 0.78	0.016
	Entropy	4.70 ± 0.87	5.40 ± 0.54	0.018
	Uniformity	0.06 ± 0.05	0.03 ± 0.01	0.015
DLE_x_	Mean (mm)	5.54 ± 2.65	7.11 ± 2.84	0.132
	Standard deviation (mm)	4.16 ± 1.94	5.51 ± 2.02	0.087
	Skewness	0.98 ± 0.49	1.10 ± 0.40	0.060
	Kurtosis	4.10 ± 2.00	4.28 ± 1.38	0.170
	Entropy	3.65 ± 0.90	4.12 ± 0.63	0.110
	Uniformity	0.11 ± 0.10	0.08 ± 0.04	0.137
DLE_y_	Mean (mm)	9.09 ± 6.14	14.49 ± 8.13	0.086
	Standard deviation (mm)	5.83 ± 3.27	8.96 ± 4.64	0.092
	Skewness	0.77 ± 0.43	0.59 ± 0.48	0.196
	Kurtosis	3.48 ± 1.17	3.28 ± 1.55	0.277
	Entropy	4.10 ± 1.05	4.84 ± 0.82	0.088
	Uniformity	0.09 ± 0.08	0.05 ± 0.03	0.098
DLE_z_	Mean (mm)	10.90 ± 6.96	16.88 ± 7.41	0.011
	Standard deviation (mm)	7.97 ± 4.62	11.89 ± 5.27	0.021
	Skewness	0.94 ± 0.43	0.71 ± 0.32	0.012
	Kurtosis	3.75 ± 1.20	3.06 ± 0.92	0.003
	Entropy	4.51 ± 0.93	5.26 ± 0.61	0.013
	Uniformity	0.06 ± 0.05	0.03 ± 0.01	0.005

Note- Data in cells indicates mean value ± standard deviation.

ILD = interstitial lung disease, DLE = degree of lung expansion. FVC = forced vital capacity, DLCO = diffusing capacity of carbon monoxide, VA = alveolar volume.

When analyzed in the upper and lower lungs, the aforementioned results of the whole lung were similar to those of the lower lung except for kurtosis (Table 2). The mean (p = 0.007 and 0.004) and 5th to 95th percentiles of the DLE_3D_ and DLE_z_ in the lower lung were significantly lower in ILD patients (Supplementary Table 5 and Supplementary Figure 1). The DLE_3D_ and DLE_z_ of the lower lung were more homogeneous in ILD patients, as shown by a smaller standard deviation (p = 0.028 and 0.048), less entropy (p = 0.020 and 0.019), and greater uniformity (p = 0.016 and 0.023). Skewness of the DLE_z_ was also significantly higher in ILD patients (p = 0.003) (Supplementary Figures 2 and 3). In the upper lung, however, the parameters were mostly similar between the two groups.

**Table 2:** Histogram parameters of DLE in upper and lower lungs in ILD patients and normal controls.

Histogram parameters		Upper		Lower		P-value	
		ILD	Normal	ILD	Normal	Upper	Lower
DLE_3D_	Mean (mm)	15.04 ± 8.56	21.26 ± 8.23	20.63 ± 10.76	32.90 ± 11.99	0.066	0.007
	Standard deviation (mm)	6.41 ± 3.52	8.72 ± 3.26	7.88 ± 3.79	10.91 ± 3.84	0.088	0.028
	Skewness	0.58 ± 0.50	0.46 ± 0.43	0.57 ± 0.39	0.44 ± 0.37	0.233	0.075
	Kurtosis	3.42 ± 1.23	3.11 ± 0.96	3.25 ± 1.02	3.08 ± 0.94	0.333	0.476
	Entropy	4.37 ± 0.93	4.97 ± 0.62	4.73 ± 0.82	5.33 ± 0.50	0.074	0.020
	Uniformity	0.07 ± 0.05	0.04 ± 0.02	0.05 ± 0.04	0.03 ± 0.01	0.087	0.016
DLE_x_	Mean (mm)	5.47 ± 2.67	6.61 ± 2.57	5.57 ± 2.99	7.67 ± 3.62	0.167	0.120
	Standard deviation (mm)	3.99 ± 1.95	5.05 ± 1.97	4.04 ± 1.98	5.71 ± 2.01	0.157	0.032
	Skewness	0.90 ± 0.55	1.05 ± 0.38	0.93 ± 0.49	1.02 ± 0.55	0.038	0.242
	Kurtosis	3.99 ± 1.99	4.22 ± 1.48	3.94 ± 2.00	4.09 ± 1.58	0.069	0.221
	Entropy	3.60 ± 0.91	3.99 ± 0.69	3.61 ± 0.91	4.17 ± 0.58	0.148	0.058
	Uniformity	0.12 ± 0.11	0.08 ± 0.05	0.12 ± 0.10	0.07 ± 0.03	0.213	0.110
DLE_y_	Mean (mm)	9.00 ± 5.97	14.49 ± 8.21	9.01 ± 6.56	14.60 ± 8.16	0.091	0.068
	Standard deviation (mm)	5.59 ± 3.28	8.57 ± 4.22	5.60 ± 3.16	9.17 ± 5.14	0.080	0.082
	Skewness	0.65 ± 0.51	0.54 ± 0.62	0.77 ± 0.51	0.53 ± 0.43	0.295	0.050
	Kurtosis	3.37 ± 1.90	3.23 ± 2.07	3.64 ± 1.48	3.12 ± 1.38	0.285	0.079
	Entropy	4.03 ± 1.10	4.78 ± 0.84	4.03 ± 0.99	4.84 ± 0.79	0.085	0.057
	Uniformity	0.10 ± 0.09	0.05 ± 0.03	0.09 ± 0.08	0.05 ± 0.03	0.112	0.055
DLE_z_	Mean (mm)	7.73 ± 5.54	9.66 ± 3.58	14.87 ± 9.42	24.47 ± 11.34	0.095	0.004
	Standard deviation (mm)	5.65 ± 4.00	6.89 ± 2.65	8.00 ± 4.46	10.88 ± 4.66	0.091	0.048
	Skewness	0.96 ± 0.44	0.88 ± 0.39	0.56 ± 0.40	0.31 ± 0.41	0.280	0.003
	Kurtosis	4.01 ± 1.50	3.76 ± 1.52	3.09 ± 0.92	2.96 ± 0.94	0.254	0.363
	Entropy	4.00 ± 0.95	4.51 ± 0.60	4.65 ± 0.90	5.28 ± 0.60	0.095	0.019
	Uniformity	0.09 ± 0.06	0.05 ± 0.02	0.06 ± 0.05	0.03 ± 0.01	0.079	0.023

Note- Data in cells indicates mean value ± standard deviation.

ILD = interstitial lung disease, DLE = degree of lung expansion.

The results of mean and standard deviation of DLE were similar after the standardization of DLE to the reference value of vital capacity (VC). In the analysis of the whole lung, the standardized mean of DLE_3D_ was significantly smaller in the ILD patients (6.44 mm/L vs. 9.90 mm/L, p = 0.033) as well as DLE_z_ (p = 0.021). The standardized standard deviation of DLE_3D_ and DLE_z_ was also significantly smaller in the ILD patients (p = 0.021 and 0.047) (Table 3). The results of the lower lung was similar to those of the whole lung, with smaller standardized mean of DLE_3D_, DLE_y_, and DLE_z_ (p = 0.010, 0.047 and 0.008) and standardized standard deviation of DLE_3D_ and DLE_x_ (p = 0.050 and 0.031). However, there was no significant difference in the standardized parameters of DLE between the two groups in the upper lung (Table 3).

**Table 3:** The standardized mean and standard deviation of DLE in ILD patients and normal controls.

Standardized histogram parameters (mm/L)		Whole lung		Upper		Lower		P-value		
		ILD	Normal	ILD	Normal	ILD	Normal	Whole lung	Upper	Lower
DLE_3D_	Mean	6.44 ± 3.37	9.90 ± 4.01	5.49 ± 2.98	7.79 ± 3.18	7.61 ± 4.05	12.16 ± 4.93	0.033	0.076	0.010
	Standard deviation	2.92 ± 1.38	4.33 ± 1.73	2.35 ± 1.21	3.19 ± 1.25	2.89 ± 1.27	4.02 ± 1.52	0.021	0.101	0.050
DLE_x_	Mean	2.05 ± 1.03	2.59 ± 1.02	2.02 ± 0.99	2.41 ± 0.95	2.09 ± 1.20	2.78 ± 1.26	0.167	0.193	0.104
	Standard deviation	1.54 ± 0.72	2.00 ± 0.73	1.47 ± 0.71	1.84 ± 0.73	1.50 ± 0.75	2.08 ± 0.72	0.094	0.176	0.031
DLE_y_	Mean	3.31 ± 2.22	5.28 ± 3.03	3.27 ± 2.15	5.29 ± 3.09	3.30 ± 2.42	5.31 ± 3.02	0.084	0.086	0.047
	Standard deviation	2.13 ± 1.16	3.26 ± 1.71	2.03 ± 1.16	3.12 ± 1.57	2.05 ± 1.15	3.32 ± 1.87	0.110	0.091	0.082
DLE_z_	Mean	4.00 ± 2.52	6.31 ± 3.06	2.82 ± 1.88	3.57 ± 1.42	5.48 ± 3.55	9.18 ± 4.67	0.021	0.104	0.008
	Standard deviation	2.92 ± 1.62	4.43 ± 2.15	2.06 ± 1.35	2.54 ± 1.06	2.93 ± 1.51	4.03 ± 1.82	0.047	0.147	0.091

Note- Data in cells indicates mean value ± standard deviation.

ILD = interstitial lung disease, DLE = degree of lung expansion.

### Correlation between inspiratory lung expansion and pulmonary function test (PFT)

Among the histogram parameters, the mean (r^2^ = 0.210; p = 0.074), entropy (r^2^ = 0.182; p = 0.100) and 5th to 95th percentiles (r^2^ = 0.194 to 0.251; p-value, 0.048 to 0.093) of the DLE_3D_ in the lower lung tended to show a weak positive correlation with forced vital capacity (FVC), and the uniformity (r^2^ = 0.210; p = 0.074) of DLE_3D_ in the lower lung showed a weak negative correlation (Table 4).

**Table 4:** Correlation between histogram parameters of DLE in the lower lung and pulmonary function test.

		FVC		%FVC		DLCO		%DLCO	
		r^2^	p-value	r^2^	p-value	r^2^	p-value	r^2^	p-value
Mean		0.210	0.074	0.103	0.226	0.069	0.342	0.056	0.395
SD		0.137	0.159	0.029	0.526	0.037	0.495	0.016	0.650
Skewness		0.000	0.995	0.103	0.226	0.032	0.523	0.179	0.116
Kurtosis		0.014	0.667	0.002	0.858	0.039	0.479	0.060	0.380
Entropy		0.182	0.100	0.045	0.429	0.023	0.589	0.012	0.692
Uniformity		0.210	0.074	0.072	0.315	0.035	0.507	0.015	0.668
Percentiles	5th	0.251	0.048	0.189	0.093	0.093	0.268	0.002	0.871
	10th	0.246	0.050	0.167	0.116	0.093	0.269	0.001	0.897
	20th	0.229	0.061	0.138	0.157	0.084	0.293	0.000	0.962
	30th	0.211	0.073	0.115	0.199	0.072	0.332	0.000	0.970
	40th	0.200	0.083	0.099	0.234	0.066	0.356	0.000	0.968
	50th	0.196	0.086	0.090	0.259	0.063	0.366	0.000	0.964
	60th	0.194	0.088	0.084	0.277	0.061	0.373	0.000	0.969
	70th	0.203	0.080	0.088	0.265	0.064	0.363	0.000	0.991
	80th	0.218	0.068	0.095	0.245	0.071	0.336	0.000	0.967
	90th	0.211	0.074	0.092	0.254	0.068	0.349	0.000	0.962
	95th	0.188	0.093	0.078	0.296	0.058	0.387	0.000	0.976

DLE = degree of lung expansion, FVC = full vital capacity, DLCO = diffusing capacity of carbon monoxide.

## Discussion

Our quantitative 3-dimensional CT analysis revealed reduced and less heterogeneous inspiratory lung expansion in ILD patients, mainly attributable to reduced craniocaudal expansion of the lower lung. These results were obtained by advanced accurate registration of paired inspiratory and expiratory chest CT images based on the surface, landmarks, and attenuation of lung parenchyma^12,13^, resulting in a mean registration error of less than 2 mm.

Little is known about the pixel-level distribution of inspiratory lung expansion in normal subjects and ILD patients. In normal subjects, the distribution of DLE_3D_ mimics an inverted U-shape with relatively preserved symmetry, and the distribution of DLE_z_ seems to be the main contributor to the shape of DLE_3D_ (Fig. 2). Based on a separate analysis of the upper and lower lungs, the distribution of DLE_3D_ and DLE_z_ in the whole lung was mainly determined by the DLE_3D_ and DLE_z_ in the lower lungs. In ILD patients, although the distribution of DLE in all 3-axes tended to be positively skewed and asymmetric, the decrease of DLE_z_ was the most pronounced (in comparison to DLE_x_ and DLE_y_), especially in the lower lungs (Fig. 2). Those differences between normal subjects and ILD patients were supported by the statistically significant decrease of the DLE_3D_ and DLE_z_ in ILD patients.

Our results also revealed less heterogeneous 3D lung expansions in ILD patients, which were especially pronounced for the lower lungs and in the craniocaudal direction. At inspiration, the lung parenchyma typically expands more in the peripheral and basal areas than in the central and upper areas^14,15^. Accordingly, the degree of peripheral and basal lung expansion is the main determinant of the degree of heterogeneity of overall lung expansion. In our ILD patients, fibrotic changes predominantly involved the basal and peripheral lungs, as in most IPF and NSIP patients^16,17^. The less heterogeneous lung expansion of ILD patients probably resulted from decreased expansion of the basal and peripheral lungs due to fibrotic changes (Fig. 1).

The reduced lung expansion in ILD patients presumably originates from reduced lung compliance due to fibrosis^18,19^. The elastic compliance of the lung parenchyma decreased in ILD patients, and the degree to which it was reduced was correlated with the degree of fibrosis due to increased resistance^20^. The increased resistance was mainly due to the abnormality of the lung parenchyma, rather than altered chest wall mechanics^19^. Although we were not able to correlate the degree of lung expansion with the degree of elastic resistance, our analysis of inspiratory lung expansion may be a noninvasive indirect imaging biomarker to assess lung compliance in ILD patients, since the elastic resistance limits the inspiratory inflation of the lung parenchyma.

The histogram parameters of lung expansion in the lower lungs were marginally correlated with FVC, which is an important predictive marker of survival in IPF patients^21,22^. Furthermore, there is no established tool to quantify inspiratory inflation of the basal lungs, where ILD is often involved. Although diaphragmatic motion can be evaluated as a proxy for basal lung movement, the compensatory lengthening and curving of the diaphragm hindered its use as an appropriate evaluation in IPF patients^23^; in contrast, our histogram analysis showed a distinct differentiation of lower lung movements between normal subjects and ILD patients. This direct quantification of lower lung movements may also be used for monitoring respiratory muscle strengthening in pulmonary rehabilitation, the importance of which is increasing in ILD patients^24^.

Our study has several limitations. First, our patient population was relatively small and exclusively consisted of women due to the paucity of normal male controls who underwent paired inspiratory and expiratory chest CT scans. Nevertheless, we expect that similar observations would be obtained in male ILD patients if there were a lower lobar predilection of fibrotic changes, as was the case in our study population. Second, it is well recognized that pulmonary function can vary with body habitus, as well as age and sex^25^. Thus, the degree of lung expansion may also be influenced by age, sex, and body habitus. Although we matched ILD patients with normal controls based on age and sex and we standardized DLE values with the reference lung volume, this may not be sufficient to precisely reflect the differences lung expansion due to age, sex, and body habitus. Therefore, further study may be required to validate our results with a sufficient number of patients, along with adjustment for age, sex, and body habitus. Third, full inspiration and end expiration might not have been sufficiently achieved during the CT scanning due to the patients’ non-compliance, although we gave detailed instructions before scanning. This might have led to an underestimation of the lung expansion between full inspiration and end expiration. Fourth, a minor portion of the destroyed peripheral lung parenchyma in ILD patients might not have been segmented due to severe fibrosis, although we adjusted the attenuation threshold values for lung segmentation in ILD patients to maximize the inclusion of fibrotic lung areas. Fifth, although we assessed regional lung expansions, our results were not based on the pulmonary lobar anatomy. However, lobar segmentation was not practically achievable in some cases due to an incomplete fissure or severe destructive fibrosis hampering a proper identification of the fissure.

In conclusion, reduced and less heterogeneous 3D inspiratory lung expansions were found in ILD patients using histogram analyses of DLE based on the advanced accurate image registration of paired inspiratory and expiratory CT scans. These results were mainly attributable to reduced craniocaudal lung expansion in the lower lung. The quantitative analysis of DLE at inspiration on chest CT scans may be a unique potential noninvasive imaging biomarker in ILD patients for indirectly evaluating elastic lung compliance and directly assessing fibrotic basal lung movements and respiratory muscle function.

## Materials and Methods

Our institutional review board (Seoul national university hospital institutional review board) approved this retrospective study, and informed consent was waived.

### Patient selection

**Figure 4:**
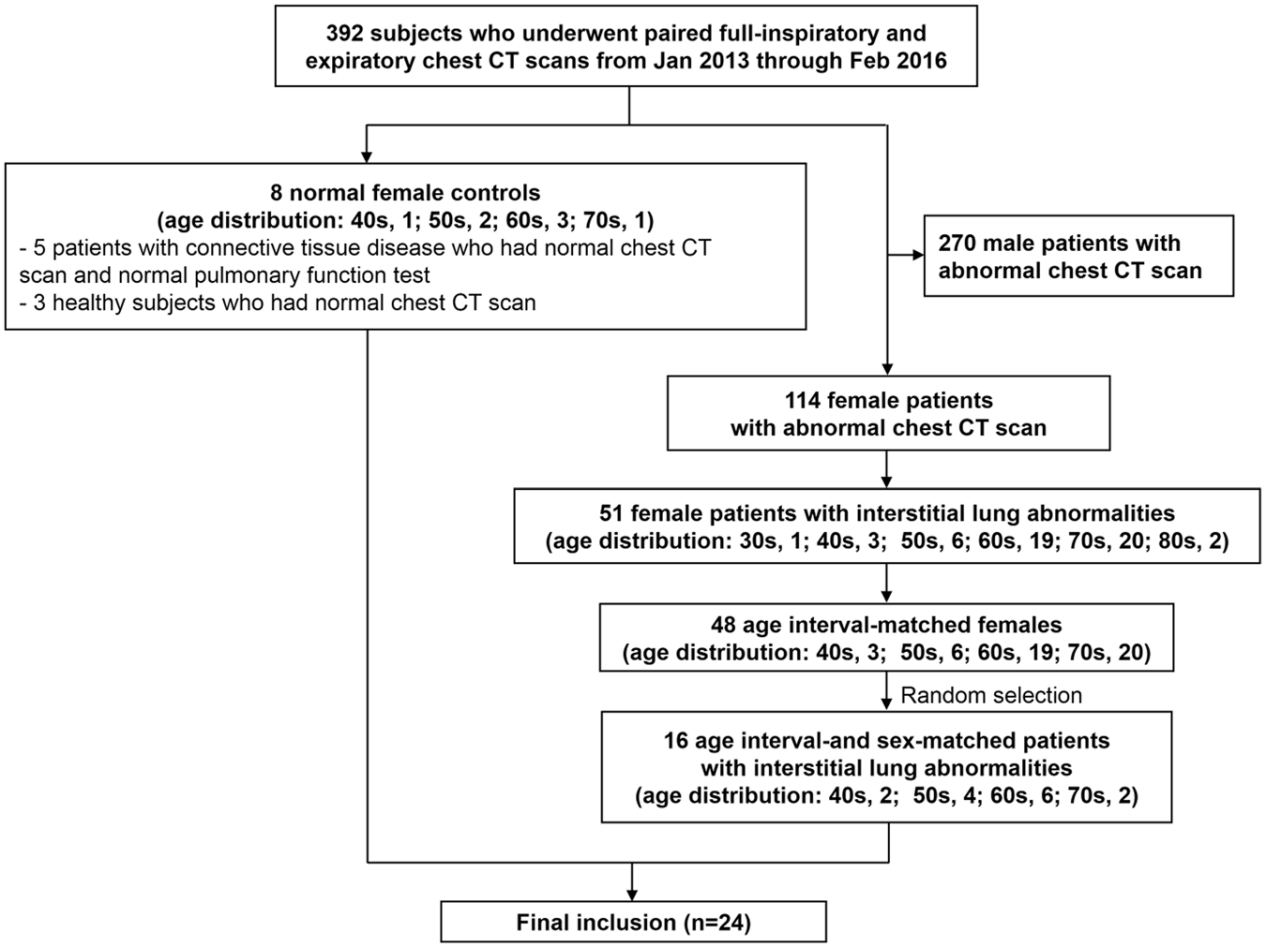
Study diagram for patient inclusion.

A total of 392 subjects who underwent paired full-inspiratory and expiratory chest CT scans from January 2013 to February 2016 were selected from the electronic medical records of our institution. Among the subjects, 8 female subjects had normal chest CT scan, and 384 had abnormal chest CT scan. All female subjects with normal chest CT scan were selected as normal female controls. As there were no normal male subjects who underwent the paired chest CT scans, we excluded male patients. Of 114 female subjects with abnormal chest CT scan, 51 subjects had interstitial lung abnormalities. 16 age interval-matched females were randomly selected within the subjects with interstitial lung abnormalities. Finally, 24 subjects (8 controls, 16 patients) were included in this study (Fig. 4).

### CT acquisition

All CT examinations were performed using multidetector CT machines with two 64-channel scanners (n = 4; Brilliance 64, Philips Medical Systems, the Netherlands) (n = 20; Ingenuity, Philips Medical Systems, the Netherlands). The scanning parameters were as follows: detector configurations, 64 × 0.625 mm; tube voltage, 120 kVp; tube current, reference mAs of 200 with automatic exposure control; pitch, 1.015; reconstruction kernel; YC0; slice thickness, 1 mm; and reconstruction interval, 1 mm. We used the standardized CT protocol for these noncontrast chest CT scans. All patients were trained in breathing prior to CT scan. Patients were instructed to perform maximal inflation so that the lung volume could be near total lung capacity (TLC) at full inspiration, and relaxed exhalation to allow the lung volume to become functional residual capacity (FRC) at end expiration as in the previous study by Kauczor H.U. *et al*.^26^. After providing detailed instructions on breathing, thin-section CT images were obtained in the supine position at full-inspiration and end expiration.

### Image segmentation and registration

**Figure 5:**
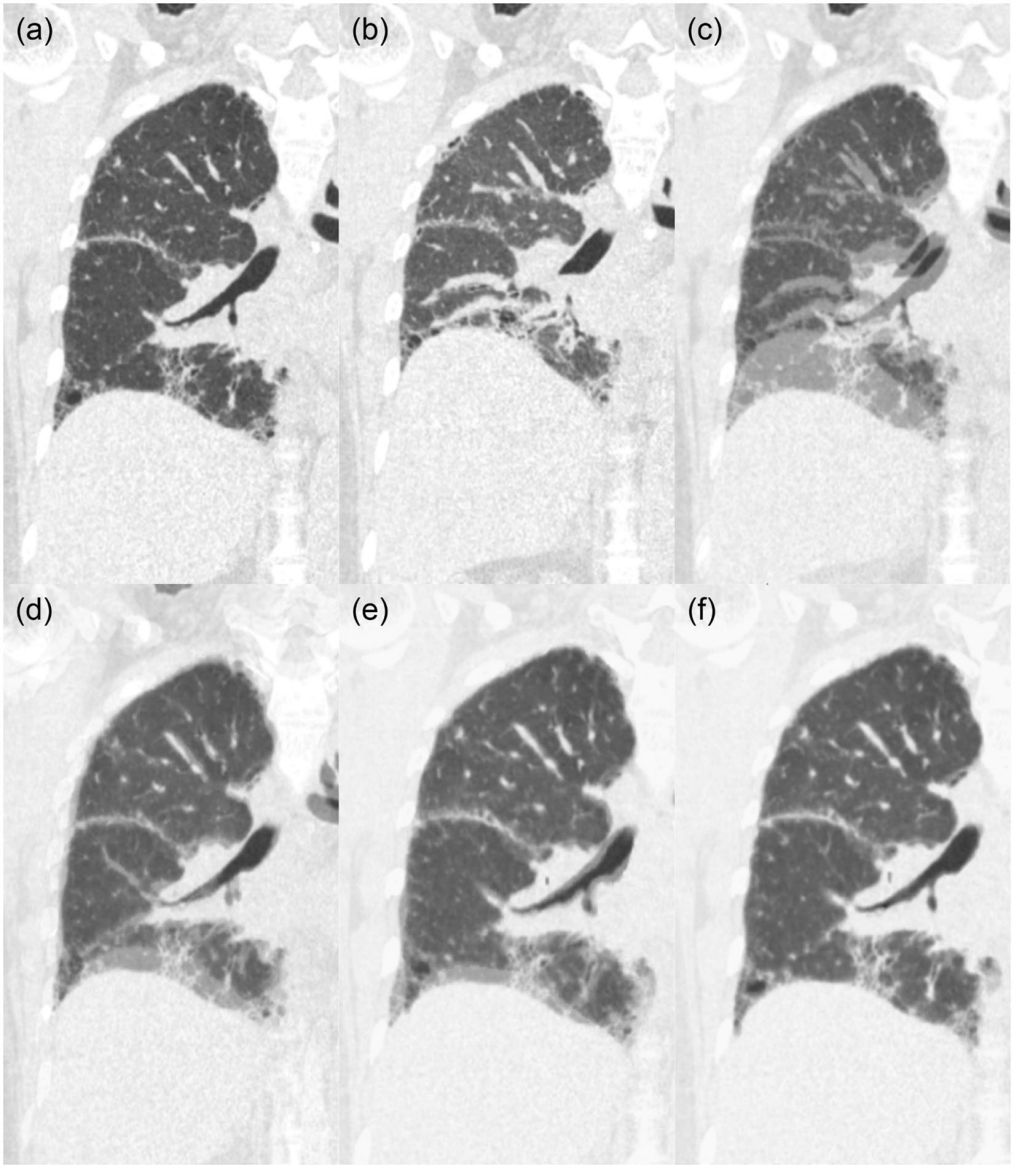
Sequential registration processes for a precise image registration of paired (**a**) full-inspiratory and (**b**) full-expiratory CT images. (**c–f**) Overlay images shows sequential inflations of a segmented lung in expiratory CT images to align with a segmented lung in inspiratory CT images: (**c**) before alignment, (**d**) after a surface-based affine registration, (**e**) landmark-based registration, and (**f**) attenuation-based deformable registration.

The paired inspiratory and expiratory CT images were aligned using in-house software (Fig. 5). To fully extract the regions of the lung containing fibrosis caused by ILD, lung segmentation was performed by one researcher (J.J.) using an appropriate threshold between −400 and −200 Hounsfield units. To check the intra-observer variability of the segmentation, the researcher repeated the segmentation after several months.

**Figure 6:**
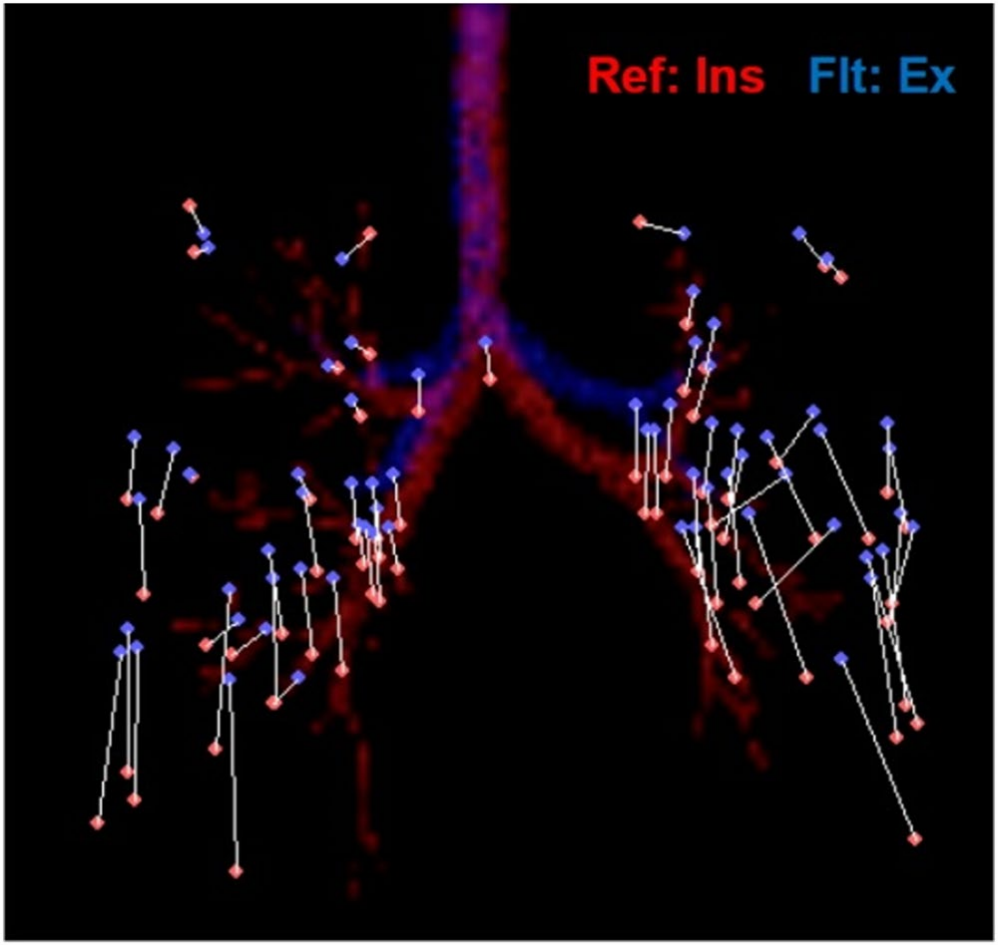
A representative map of landmarks of the bronchial tree and pulmonary vessels. In this patient, a total of 67 landmarks (33 in right lung, 33 in left lung, and on at carina) were placed in a side-by-side display of 1-mm-thick inspiratory and expiratory CT images. The landmarks in the inspiratory (red dots) and expiratory CT (blue dots) are paired to represent the same location.

The segmented lung in the expiratory CT image (source image) was then globally aligned with the segmented lung in the inspiratory CT image (target image) by inflating the expiratory lung using surface-based affine registration^12^. To improve the alignment of the internal structures of the lung parenchyma, landmark-based registration using a thin-plate spline warping was performed: one radiologist chose 31–34 landmarks of the bronchial tree and pulmonary vessel per lung^13^. The landmarks were marked carefully by placing small dots in the side-by-side display of 1-mm-thick inspiratory and expiratory CT images to ensure that identical locations were annotated in the CT images. The branching points of the lobar and segmental bronchi served as the bronchial landmarks, while pulmonary vessels located within 2 cm from the pleura were used for the vascular landmarks. The landmarks were evenly distributed in the upper to lower lungs (Fig. 6). Finally, the registered lungs were locally aligned using attenuation-based demons deformable registration^12^. The main idea of the demons deformable registration is to position ‘demons’ at certain voxels of the target image and to make the source image diffuse to the target image using the estimated force based on the difference in attenuation between the target and source images.

### Accuracy evaluation of image registration

To quantitatively evaluate the accuracy of image registration, we calculated the registration error for each patient as the mean distance (mm) of the corresponding bronchial and vascular landmarks between the inspiratory CT and registered expiratory CT images. The registration error was assessed at each step of applying three registration methods (surface-based affine registration, landmark-based registration, and attenuation-based deformable registration) in order to identify the effect of combining the registration approaches, especially for landmark-based registration.

### Inspiratory lung expansion analysis

After checking the accuracy of image registration between the inspiratory and expiratory CT scans, we measured DLE of each pixel between the two CT scans in the following axes: horizontal axis (x-axis; DLE_x_), ventrodorsal axis (y-axis; DLE_y_), craniocaudal axis (z-axis; DLE_z_), and 3-dimensionally (3D; DLE_3D_). Histogram analyses were then performed for the right and left lungs in all axes. Each lung was divided into the upper and lower lungs at the branching point of the lower lobar bronchus from either the right bronchus intermedius or the left main bronchus. We performed the analyses for the whole lung and for the upper and lower lungs separately.

The following histogram parameters were calculated using the probability distribution of the DLEs in each axis: mean, standard deviation, skewness, kurtosis, and the 5th, 10th, 20th, …, 90th and 95th percentiles, entropy and uniformity.

The mean (M) is the average value, and is calculated as equation (1):

1 $${\rm{M}}=\sum _{{\rm{i}}=1}^{{\rm{N}}}{{\rm{x}}}_{{\rm{i}}}{\rm{P}}({{\rm{x}}}_{{\rm{i}}})$$

where N is the number of discrete bins of the histogram and P(x_i_) is the probability of bin x_i_.

The standard deviation (SD), which is a measure of contrast, is calculated as equation (2); it describes the spread in the data, so a high contrast in DLE will have high variance and a low contrast in DLE will have low variance.

2 $${\rm{SD}}=\sqrt{\sum _{{\rm{i}}=1}^{{\rm{N}}}{({{\rm{x}}}_{{\rm{i}}}-{\rm{M}})}^{2}{\rm{P}}({{\rm{x}}}_{{\rm{i}}})}$$

Skewness (SK), which is a measure of asymmetry about the mean in the DLE distribution, is calculated as equation (3); it is positive if the tail of the histogram spreads to the right, and negative if the tail of the histogram spreads to the left.

3 $${\rm{SK}}=\frac{1}{{{\rm{SD}}}^{3}}\sum _{{\rm{i}}=1}^{{\rm{N}}}{({{\rm{x}}}_{{\rm{i}}}-{\rm{M}})}^{3}{\rm{P}}({{\rm{x}}}_{{\rm{i}}})$$

Kurtosis (K), which is a measure of the relative flatness of the histogram, is calculated as equation (4); a high value of kurtosis indicates that the peak of the distribution is sharp and the tail is longer and fat. A low value of kurtosis indicates that the peak of the distribution is rounded and the tail is shorter and thinner.

4 $${\rm{K}}=\frac{1}{{{\rm{SD}}}^{4}}\sum _{{\rm{i}}=1}^{{\rm{N}}}{({{\rm{x}}}_{{\rm{i}}}-{\rm{M}})}^{4}{\rm{P}}({{\rm{x}}}_{{\rm{i}}})$$

Entropy (E), which is a measure of the variability of the DLE distribution, is calculated as equation (5), and increases as the variability of P(x_i_) increases.

5 $${\rm{E}}=-\sum _{{\rm{i}}=1}^{{\rm{N}}}{\rm{P}}({{\rm{x}}}_{{\rm{i}}}){\rm{logP}}({{\rm{x}}}_{{\rm{i}}})$$

Uniformity (U), which is a measure of the homogeneity of the DLE distribution, is defined as equation (6), and is maximized when all P(x_i_) are equal.

6 $${\rm{U}}=\sum _{{\rm{i}}=1}^{{\rm{N}}}{{\rm{P}}}^{2}({{\rm{x}}}_{{\rm{i}}})$$

Since the DLE increases with increasing lung volume, we calculated standardized mean and standard deviation of DLE in each axis by dividing the values by the reference value of VC to adjust the differences of DLE according to the reference lung volume. The reference lung volume was calculated according to the previous study^27^, and the reference value of VC was calculated by subtracting the reference value of RV from TLC. The standardized parameters of DLE were separately compared between the ILD patients and the normal controls.

### CT visual analysis

Visual analysis was performed by a resident (J.H.P.) from the Department of Radiology under the supervision of an experienced thoracic radiologist (S.H.Y., 12 years of thoracic CT experience) to assess the degree of pulmonary fibrosis. The mean extent of fibrotic changes, including reticular opacity and honeycombing, was scored at 5% intervals in the upper, middle, and lower zones of each lung using the inspiratory CT images. Each zone was defined according to the previous study by Best *et al*.^8^. The degree of pulmonary fibrosis was summarized as the mean value of the fibrosis score of the three zones.

### Correlation with pulmonary function tests

Sixteen of the 24 subjects underwent a PFT within four weeks of chest CT scanning. The mean interval between the PFT and CT scanning was 4.4 ± 7.9 days (range, 0–26 days). We obtained FVC, diffusing capacity of carbon monoxide (DLCO) and alveolar volume (VA) values in the PFT. Results were expressed as both absolute value (FVC and DLCO) and percentage of predicted performance (%FVC and %DLCO).

### Statistical analysis

The Dice similarity coefficient was used to calculate the intra-observer agreement of the segmentation process^28^. Nonparametric repeated-measures analysis of variance was used to compare the histogram parameters of DLE in each axis between the ILD patients and the normal controls. The Pearson correlation coefficient or Spearman correlation coefficient by rank was used to assess the relationship between PFT results and the histogram features of DLE_3D_ in the lower lung depending on whether or not they showed a normal distribution. A two-tailed p-value of less than 0.05 was considered to indicate a significant difference. Statistical analyses were carried out using SAS software (version 9.4; SAS Institute Inc., Cary, NC, USA) and SPSS software (version 23; IBM Corp., Armonk, NY, USA).

## Electronic supplementary material

Supplementary tables and figures

## Data Availability

All data generated or analyzed during this study are included in this published article and its Supplementary Information files.
