# Author Correction: Inspiratory Lung Expansion in Patients with Interstitial Lung Disease: CT Histogram Analyses

**DOI:** 10.1038/s41598-019-55813-4

**Published:** 2019-12-11

**Authors:** Junghoan Park, Julip Jung, Soon Ho Yoon, Jin Mo Goo, Helen Hong, Jeong-Hwa Yoon

**Affiliations:** 1 Department of Radiology, Seoul National University Hospital, Seoul, Korea; 2 Department of Radiology, Seoul National University College of Medicine, Seoul, Korea; 3 Department of Software Convergence, Seoul Women’s University, Seoul, Korea; 4 Institute of Radiation Medicine, Seoul National University Medical Research Center, Seoul, Korea; 5 Interdisciplinary Program in Medical Informatics, Seoul National University College of Medicine, Seoul, Korea

Correction to: *Scientific Report* 10.1038/s41598-018-33638-x, published online 15 October 2018

The Acknowledgements section in this Article was omitted. The Acknowledgements section should read:

“This work was supported by the National Research Foundation of Korea (NRF) grant funded by the Korea government (MSIT) (2016R1C1B1015761).”

