# Supplementary material for: Inspiratory Lung Expansion in Patients with Interstitial Lung Disease: CT Histogram Analyses
Source: Sci Rep. 2018 Oct 15;8:15265. doi: 10.1038/s41598-018-33638-x (PMC6189065; doi:10.1038/s41598-018-33638-x)

**Inspiratory Lung Expansion in Patients with Interstitial Lung Disease: CT Histogram Analyses**

Junghoan Park M.D.^1,2†^, Julip Jung Ph.D.^3†^, Soon Ho Yoon, M.D. (*)^1,2^, Jin Mo Goo, M.D., Ph.D.^1,2,4^, Helen Hong, Ph.D.^3^, Jeong-Hwa Yoon, M.S.^5^

^1^Department of Radiology, Seoul National University Hospital, Seoul, Korea;

^2^Department of Radiology, Seoul National University College of Medicine, Seoul, Korea;

^3^Department of Software Convergence, Seoul Women’s University, Seoul, Korea;

^4^Institute of Radiation Medicine, Seoul National University Medical Research Center, Seoul, Korea;

^5^Interdisciplinary Program in Medical Informatics, Seoul National University College of Medicine, Seoul, Korea

†These two authors contributed equally to this work

**Supplemental materials**

**Supplementary table 1.** The Dice similarity coefficient between repeated lung segmentation by the same researcher

|  | Inspiratory CT | | Expiratory CT | |
| --- | --- | --- | --- | --- |
|  | Right | Left | Right | Left |
| ILD | 0.991 (0.959 - 0.999) | 0.989 (0.962 - 0.999) | 0.966 (0.900 - 0.997) | 0.949 (0.814 - 0.999) |
| Normal | 0.980 (0.964 - 0.998) | 0.967 (0.946 - 0.996) | 0.973 (0.950 - 1.000) | 0.955 (0.906 - 1.000) |
| All images | 0.972 (0.814 - 1.000) | | | |

Note- Data in cells indicates mean (minimum – maximum) of the Dice similarity coefficient

ILD = interstitial lung disease

**Supplementary table 2.** Registration error in sequential processes of image registration of inspiratory and expiratory chest CT scans

| Rt. Lung | | | | | |
| --- | --- | --- | --- | --- | --- |
| (mm) | Without registration | Affine registration | Affine  + Deformable | Affine  + Bronchial TPS  + Deformable | Affine + Bronchial TPS + Pph. vessel TPS + Deformable |
| Overall | 13.1 ± 6.7 | 8.2 ± 6.0 | 5.7 ± 4.7 | 4.1 ± 2.9 | 1.9 ± 1.2 |
| ILD | 11.2 ± 5.5 | 5.9 ± 5.2 | 3.5 ± 3.6 | 3.3 ± 2.9 | 1.5 ± 1.0 |
| Normal | 17.1 ± 7.3 | 12.6 ± 5.3 | 10.1 ± 3.5 | 5.7 ± 2.5 | 2.8 ± 1.3 |
| Lt. lung | | | | | |
| (mm) | Without registration | Affine registration | Affine  + Deformable | Affine  + Bronchial TPS  + Deformable | Affine + Bronchial TPS + Pph. vessel TPS + Deformable |
| Overall | 15.6 ± 8.7 | 7.5 ± 6.5 | 7.7 ± 7.4 | 4.8 ± 2.9 | 1.9 ± 0.9 |
| ILD | 13.6 ± 8.0 | 6.8 ± 6.9 | 6.5 ± 7.6 | 4.7 ± 3.2 | 1.8 ± 0.8 |
| Normal | 21.0 ± 9.0 | 9.8 ± 5.8 | 11.0 ± 6.4 | 5.1 ± 2.4 | 2.4 ± 1.2 |

Note- Data in cells indicates mean value ± standard deviation.

Bronchial TPS: Landmark-based registration using landmarks of the bronchial tree

Pph. vessel TPS: Landmark-based registration using landmarks of the pulmonary vessel

**Supplementary table 3.** The mean lung volume differences between inspiratory and registered expiratory lung in sequential processes of image registration.

| **Rt. Lung** | | | | |
| --- | --- | --- | --- | --- |
| **Volume difference (ml)** | Without registration | Affine registration | Affine + TPS | Affine + TPS + Deformable |
| **Overall** | 484.2 ± 206.9 | 239.6 ± 151.9 | 145.5 ± 106.8 | 91.6 ± 81.3 |
| **ILD** | 446.2 ± 223.0 | 207.2 ± 143.7 | 111.5 ± 78.4 | 63.8 ± 55.3 |
| **Normal** | 560.3 ± 155.6 | 304.3 ± 156.2 | 213.3 ± 128.2 | 147.1 ± 99.4 |
| **Lt. lung** | | | | |
| **Volume difference (ml)** | Without registration | Affine registration | Affine + TPS | Affine + TPS + Deformable |
| **Overall** | 421.2 ± 229.5 | 257.0 ± 195.4 | 182.0 ± 139.7 | 99.9 ± 93.6 |
| **ILD** | 348.3 ± 234.3 | 214.8 ± 186.6 | 163.8 ± 144.7 | 82.7 ± 84.5 |
| **Normal** | 567.0 ± 137.9 | 341.3 ± 196.6 | 218.4 ± 130.2 | 134.2 ± 106.9 |

Note - Data in cells indicates mean value ± standard deviation.

Volume difference = Inspiratory lung volume – registered expiratory lung volume

TPS = landmark-based registration using a thin-plate spline warping

**Supplementary table 4.** Comparisons of percentile values of DLE between ILD and normal control groups

| Percentile values |  | ILD  (n=16) | Normal control  (n=8) | | P-value |
| --- | --- | --- | --- | --- | --- |
| DLE_3D_ | 5th | 6.40±3.64 | 10.06±4.43 | | 0.049 |
|  | 10th | 8.16±4.62 | 12.85±5.45 | | 0.033 |
|  | 20th | 10.69±6.08 | 16.66±6.98 | | 0.037 |
|  | 30th | 12.79±7.38 | 19.67±8.17 | | 0.034 |
|  | 40th | 14.70±8.51 | 22.46±9.27 | | 0.038 |
|  | 50th | 16.59±9.50 | 25.40±10.25 | | 0.031 |
|  | 60th | 18.64±10.47 | 28.56±10.95 | | 0.026 |
|  | 70th | 20.95±11.38 | 32.17±11.67 | | 0.027 |
|  | 80th | 23.96±12.58 | 36.84±12.91 | | 0.021 |
|  | 90th | 28.42±14.27 | 43.19±14.89 | | 0.015 |
|  | 95th | 32.19±15.67 | 48.07±16.63 | | 0.020 |
| DLE_x_ | 5th | 0.44±0.32 | 0.49±0.28 | | 0.519 |
|  | 10th | 0.86±0.57 | 0.98±0.56 | | 0.522 |
|  | 20th | 1.65±1.00 | 1.95±1.10 | | 0.481 |
|  | 30th | 2.46±1.42 | 2.96±1.63 | | 0.429 |
|  | 40th | 3.29±1.84 | 4.03±2.17 | | 0.374 |
|  | 50th | 4.18±2.26 | 5.20±2.73 | | 0.345 |
|  | 60th | 5.15±2.70 | 6.51±3.34 | | 0.284 |
|  | 70th | 6.29±3.21 | 8.03±3.97 | | 0.271 |
|  | 80th | 7.72±3.79 | 9.91±4.65 | | 0.226 |
|  | 90th | 9.87±4.64 | 12.70±5.46 | | 0.239 |
|  | 95th | 11.87±5.45 | 15.22±6.08 | | 0.184 |
| DLE_y_ | 5th | 1.21±1.68 | 1.68±1.39 | | 0.115 |
|  | 10th | 2.08±2.26 | 3.09±2.22 | | 0.119 |
|  | 20th | 3.68±3.22 | 5.76±3.67 | | 0.115 |
|  | 30th | 5.26±4.24 | 8.33±5.12 | | 0.103 |
|  | 40th | 6.79±5.19 | 10.94±6.70 | | 0.093 |
|  | 50th | 8.33±6.12 | 13.56±8.30 | | 0.100 |
|  | 60th | 9.91±7.03 | 16.16±9.87 | | 0.109 |
|  | 70th | 11.63±8.01 | 18.90±11.48 | | 0.117 |
|  | 80th | 13.59±9.01 | 22.03±13.24 | | 0.112 |
|  | 90th | 16.23±10.15 | 25.77±15.03 | | 0.118 |
|  | 95th | 18.34±10.94 | 28.39±16.00 | | 0.126 |
| DLE_z_ | 5th | 1.09±0.91 | 1.45±0.70 | | 0.067 |
|  | 10th | 2.04±1.60 | 2.84±1.33 | | 0.055 |
|  | 20th | 3.81±2.83 | 5.56±2.60 | | 0.038 |
|  | 30th | 5.54±4.04 | | 8.30±3.87 | 0.031 |
|  | 40th | 7.31±5.28 | | 11.18±5.17 | 0.021 |
|  | 50th | 9.29±6.63 | | 14.35±6.59 | 0.020 |
|  | 60th | 11.51±7.93 | | 17.81±7.82 | 0.011 |
|  | 70th | 14.07±9.19 | | 21.83±9.26 | 0.009 |
|  | 80th | 17.23±10.77 | | 26.63±11.13 | 0.006 |
|  | 90th | 21.79±13.05 | | 32.68±13.44 | 0.010 |
|  | 95th | 25.30±14.64 | | 37.43±15.56 | 0.020 |

Note- Data in cells indicate mean value ± standard deviation.

ILD = interstitial lung disease, DLE = degree of lung expansion.

**Supplementary table 5.** Comparisons of percentile values of DLE in upper and lower lungs between ILD and normal control groups

|  |  | Upper | | Lower | | P-value | |
| --- | --- | --- | --- | --- | --- | --- | --- |
| Percentile values | | **ILD** | **Normal** | **ILD** | **Normal** | **Upper** | **Lower** |
| DLE_3D_ | 5th | 5.58±3.50 | 8.34±3.83 | 9.30±6.68 | 16.97±7.83 | 0.084 | 0.002 |
|  | 10th | 7.10±4.36 | 10.45±4.42 | 11.07±7.41 | 19.76±8.61 | 0.065 | 0.002 |
|  | 20th | 9.35±5.71 | 13.55±5.49 | 13.55±8.43 | 23.42±9.69 | 0.050 | 0.003 |
|  | 30th | 11.13±6.75 | 16.04±6.44 | 15.63±9.24 | 26.29±10.40 | 0.042 | 0.004 |
|  | 40th | 12.73±7.68 | 18.26±7.32 | 17.63±9.99 | 28.96±11.01 | 0.044 | 0.005 |
|  | 50th | 14.34±8.56 | 20.45±8.21 | 19.65±10.75 | 31.70±11.60 | 0.056 | 0.006 |
|  | 60th | 16.08±9.46 | 22.80±9.19 | 21.82±11.63 | 34.69±12.31 | 0.071 | 0.005 |
|  | 70th | 17.96±10.34 | 25.46±10.25 | 24.19±12.49 | 38.15±13.31 | 0.087 | 0.006 |
|  | 80th | 20.20±11.36 | 28.78±11.33 | 26.97±13.37 | 42.36±14.66 | 0.085 | 0.008 |
|  | 90th | 23.59±12.96 | 33.23±12.60 | 31.07±14.82 | 47.78±16.50 | 0.070 | 0.010 |
|  | 95th | 26.53±14.40 | 36.63±13.38 | 34.82±16.58 | 52.07±18.34 | 0.063 | 0.012 |
| DLE_x_ | 5th | 0.45±0.33 | 0.47±0.25 | 0.52±0.48 | 0.61±0.55 | 0.530 | 0.240 |
|  | 10th | 0.87±0.59 | 0.93±0.49 | 0.99±0.84 | 1.23±1.11 | 0.544 | 0.248 |
|  | 20th | 1.66±1.02 | 1.85±0.96 | 1.87±1.42 | 2.40±2.12 | 0.496 | 0.301 |
|  | 30th | 2.45±1.42 | 2.78±1.42 | 2.72±1.91 | 3.50±2.86 | 0.477 | 0.281 |
|  | 40th | 3.25±1.79 | 3.77±1.86 | 3.56±2.35 | 4.63±3.49 | 0.417 | 0.239 |
|  | 50th | 4.07±2.15 | 4.82±2.31 | 4.44±2.80 | 5.84±4.03 | 0.409 | 0.194 |
|  | 60th | 4.99±2.56 | 5.97±2.80 | 5.42±3.26 | 7.21±4.52 | 0.379 | 0.175 |
|  | 70th | 6.05±3.00 | 7.31±3.36 | 6.57±3.80 | 8.81±5.00 | 0.344 | 0.147 |
|  | 80th | 7.37±3.52 | 8.96±4.04 | 8.03±4.49 | 10.77±5.49 | 0.378 | 0.127 |
|  | 90th | 9.36±4.31 | 11.46±4.90 | 10.15±5.48 | 13.57±5.98 | 0.369 | 0.113 |
|  | 95th | 11.16±5.11 | 13.73±5.55 | 11.88±6.14 | 16.01±6.23 | 0.265 | 0.089 |
| DLE_y_ | 5th | 1.14±1.32 | 2.02±1.97 | 1.52±2.57 | 1.54±1.11 | 0.125 | 0.111 |
|  | 10th | 2.02±1.86 | 3.50±2.82 | 2.40±3.15 | 2.93±1.92 | 0.126 | 0.085 |
|  | 20th | 3.64±2.84 | 6.14±4.25 | 4.00±4.14 | 5.58±3.39 | 0.133 | 0.072 |
|  | 30th | 5.22±3.83 | 8.57±5.57 | 5.47±5.02 | 8.22±4.85 | 0.150 | 0.065 |
|  | 40th | 6.79±4.83 | 10.99±6.95 | 6.93±5.94 | 11.07±6.69 | 0.144 | 0.067 |
|  | 50th | 8.37±5.83 | 13.45±8.36 | 8.42±6.77 | 13.79±8.44 | 0.169 | 0.073 |
|  | 60th | 9.96±6.80 | 15.99±9.76 | 10.06±7.64 | 16.49±10.13 | 0.148 | 0.059 |
|  | 70th | 11.60±7.73 | 18.67±11.15 | 11.79±8.59 | 19.45±12.14 | 0.137 | 0.077 |
|  | 80th | 13.41±8.67 | 21.59±12.54 | 13.73±9.63 | 22.67±14.25 | 0.112 | 0.090 |
|  | 90th | 15.69±9.71 | 25.05±14.08 | 16.12±10.60 | 26.33±15.96 | 0.123 | 0.100 |
|  | 95th | 17.45±10.45 | 27.47±14.99 | 17.94±11.08 | 28.71±16.69 | 0.127 | 0.085 |
| DLE_z_ | 5th | 0.74±0.65 | 0.81±0.39 | 4.29±5.98 | 9.15±7.03 | 0.270 | < 0.001 |
|  | 10th | 1.42±1.15 | 1.60±0.74 | 5.80±6.70 | 11.65±7.49 | 0.273 | < 0.001 |
|  | 20th | 2.67±2.05 | 3.13±1.35 | 8.09±7.52 | 15.25±7.91 | 0.233 | < 0.001 |
|  | 30th | 3.88±2.93 | 4.64±1.94 | 10.08±8.10 | 18.16±8.40 | 0.175 | < 0.001 |
|  | 40th | 5.08±3.80 | 6.20±2.56 | 12.03±8.69 | 20.94±9.11 | 0.150 | < 0.001 |
|  | 50th | 6.33±4.71 | 7.81±3.19 | 14.03±9.37 | 23.73±9.94 | 0.121 | 0.001 |
|  | 60th | 7.73±5.72 | 9.54±3.84 | 16.11±10.14 | 26.56±10.94 | 0.141 | 0.002 |
|  | 70th | 9.35±6.89 | 11.46±4.52 | 18.38±11.02 | 29.53±12.05 | 0.118 | 0.004 |
|  | 80th | 11.45±8.45 | 13.85±5.33 | 20.95±11.97 | 32.89±13.35 | 0.128 | 0.006 |
|  | 90th | 14.56±10.89 | 17.41±6.39 | 24.81±13.82 | 37.61±15.48 | 0.117 | 0.012 |
|  | 95th | 17.11±12.46 | 20.42±7.31 | 28.20±15.85 | 41.50±17.52 | 0.101 | 0.023 |

Note- Data in cells indicate mean value ± standard deviation.

ILD = interstitial lung disease, DLE = degree of lung expansion.

Supplementary figure 1. Graphs representing 5th to 95th percentile values of degree of lung expansion in upper (a) and lower (b) lungs in 3-dimensional axis, x-axis, y-axis, z-axis. A box with a black line indicates statistically significant difference between two groups (p < .05).


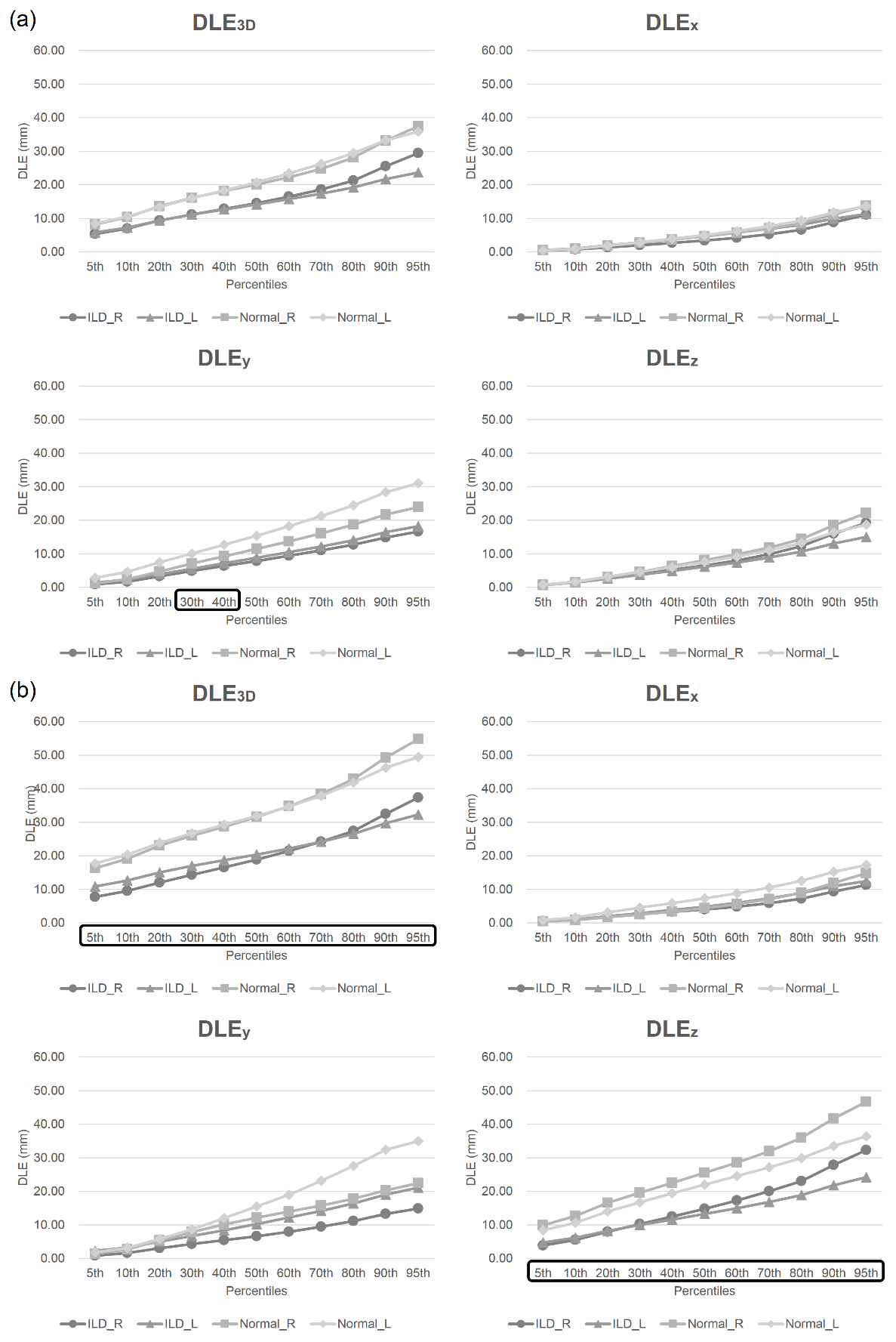


Supplementary figure 2. Histogram of degree of upper lung expansion in patients with interstitial lung disease patients (blue) and normal control (orange) in (a) 3-dimensional, and (b) x-, (c) y-, (d) z-axes.


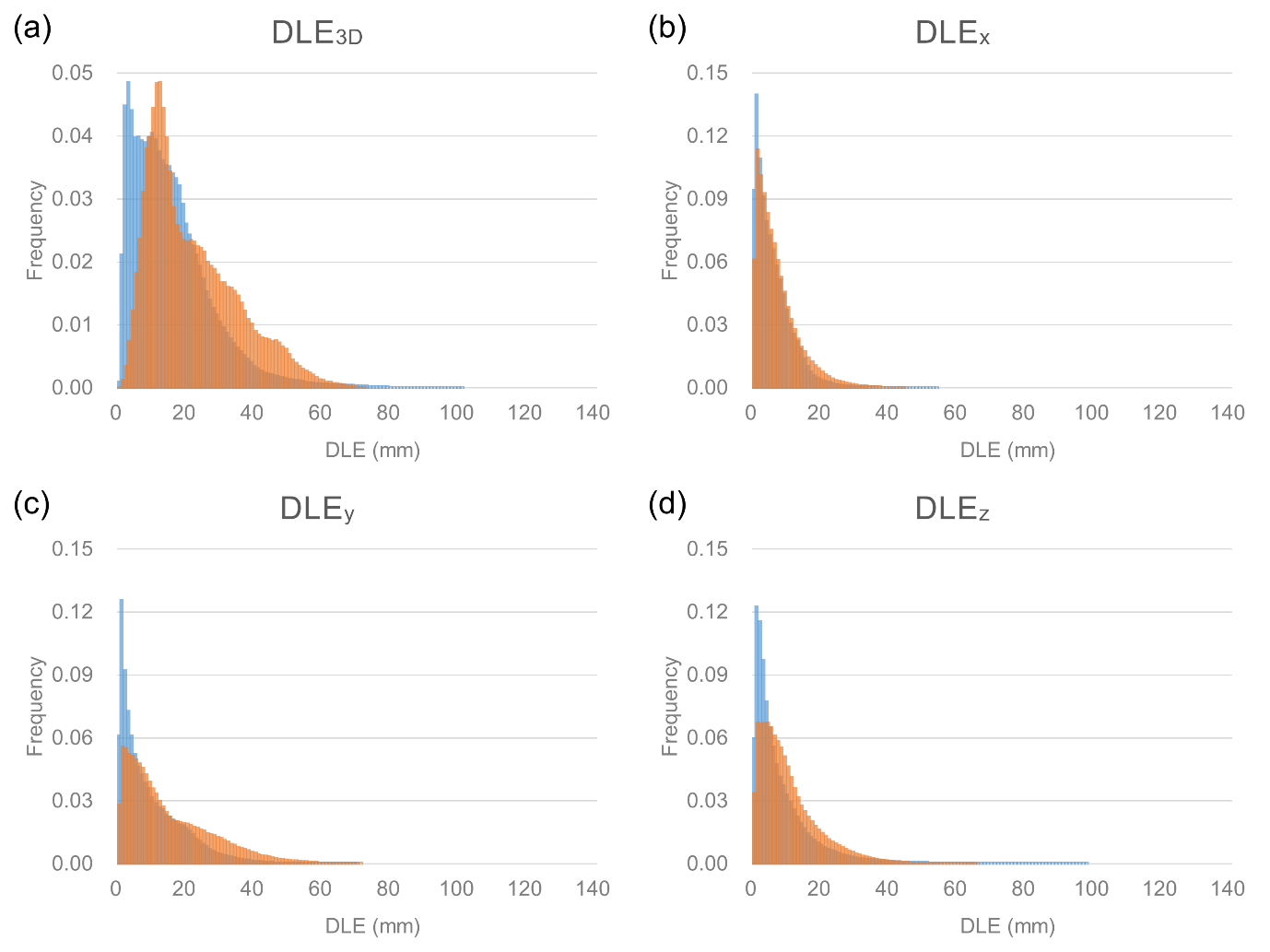


Supplementary figure 3. Histogram of degree of lower lung expansion in patients with interstitial lung disease patients (blue) and normal control (orange) in (a) 3-dimensional, and (b) x-, (c) y-, (d) z-axes.


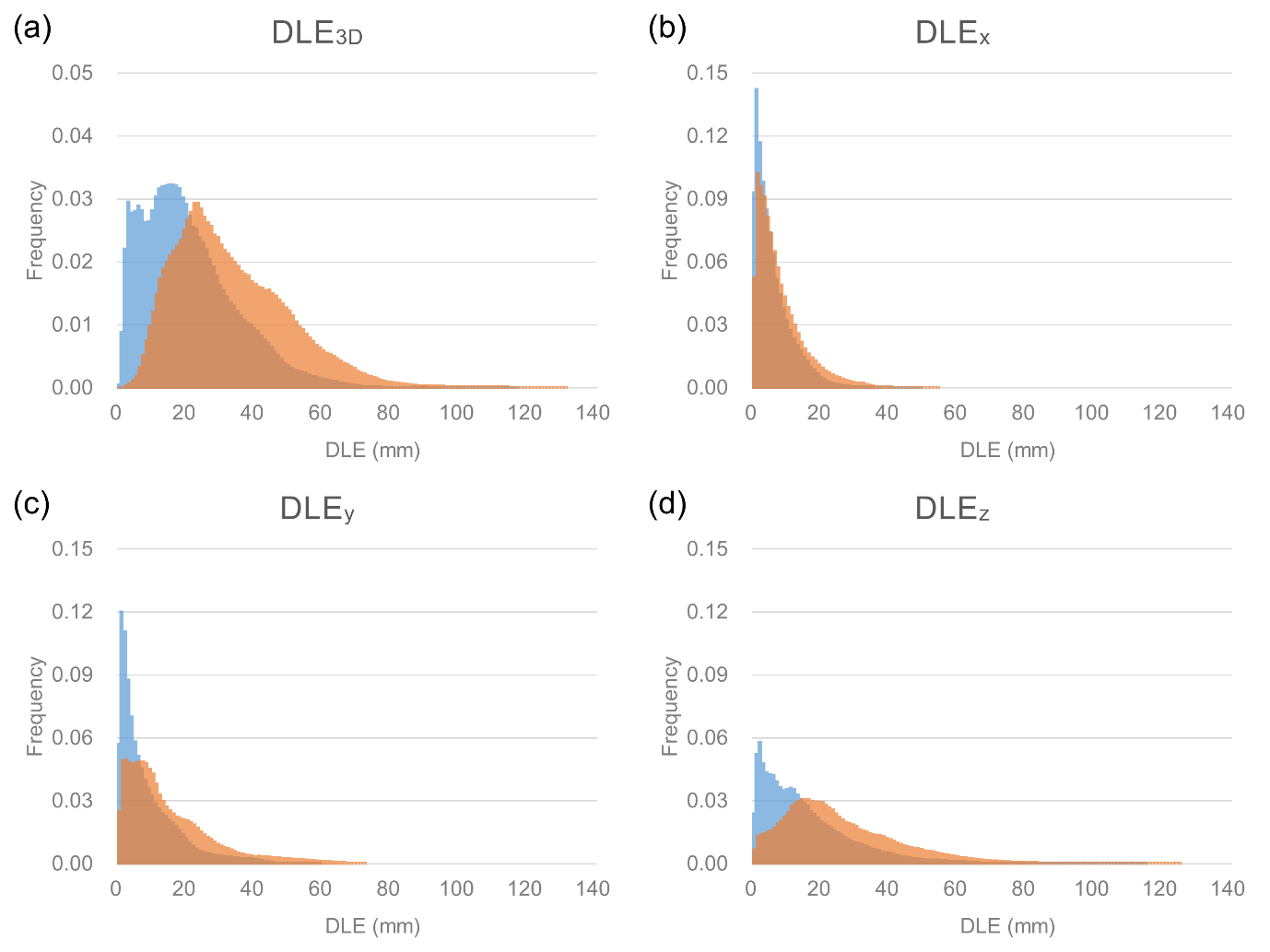

Supplement: Supplementary file 1 — Supplementary tables and figures [file 41598_2018_33638_MOESM1_ESM.docx]
